# Control systems theory revisited: new insights on the brain clocks of time-to-action

**DOI:** 10.3389/fnins.2023.1171765

**Published:** 2023-06-09

**Authors:** Sari Goldstein Ferber, Aron Weller, Hermona Soreq

**Affiliations:** ^1^Department of Psychology, Gonda Brain Research Center, Bar-Ilan University, Ramat Gan, Israel; ^2^Department of Psychology and Brain Sciences, University of Delaware, Newark, DE, United States; ^3^The Edmond and Lily Safra Center for Brain Sciences, The Alexander Silberman Institute of Life Sciences, The Hebrew University of Jerusalem, Jerusalem, Israel

**Keywords:** brain timescales, goal-oriented behavior, spatiotemporal regulation, biological clocks/physiology, control systems

## Abstract

To outline the complex biological rhythms underlying the time-to-action of goal-oriented behavior in the adult brain, we employed a Boolean Algebra model based on Control Systems Theory. This suggested that “timers” of the brain reflect a metabolic excitation-inhibition balance and that healthy clocks underlying goal-oriented behavior (optimal range of signal variability) are maintained by XOR logic gates in parallel sequences between cerebral levels. Using truth tables, we found that XOR logic gates reflect healthy, regulated time-to-action events between levels. We argue that the brain clocks of time-to-action are active within multileveled, parallel-sequence complexes shaped by experience. We show the metabolic components of time-to-action in levels ranging from the atom level through molecular, cellular, network and inter-regional levels, operating as parallel sequences. We employ a thermodynamic perspective, suggest that clock genes calculate free energy versus entropy and derived time-to-action level-wise as a master controller, and show that they are receivers, as well as transmitters of information. We argue that regulated multileveled time-to-action processes correspond to Boltzmann’s thermodynamic theorem of micro- and macro-states, and that the available metabolic free-energy-entropy matrix determines the brain’s reversible states for its age-appropriate chrono-properties at given moments. Thus, healthy timescales are not a precise number of nano- or milliseconds of activity nor a simple phenotypic distinction between slow vs. quick time-to-action, but rather encompass a range of variability, which depends on the molecules’ size and dynamics with the composition of receptors, protein and RNA isoforms.

## Introduction

Goal-oriented behaviors are the seminal component of decision-making, vital for survival and exhibited in a time-dependent manner, reflecting the brain’s chrono-properties (Honma, 2020), which have recently gained increased attention (Gupta et al., 2020). Cells sensitive to time differences were identified in several brain regions (MacDonald et al., 2011), and much interest has been focused on the temporal aspects underlying overt behavior. Together, this suggests a complex view beyond the accepted measure of behavioral reaction time and the simple discrimination between rapid and slow decision-making.

Control Systems Theory (CST), initially described mathematically by Maxwell in the 19th century, underwent major developments since then (e.g., Lyshevski, 2001; Schiff, 2011). This theory implies dynamic control of signals by internal controllers implemented in the system, to attain a required level of a signal (set point) compared to an actual level of that signal. Following that comparison, the controller produces a corrected output. Consistent feedback comparisons are hence an essential part of CST and are applicable for the behavioral outcomes, as outputs of the CNS (Bennett, 1993; Goldstein Ferber et al., 2022). To avoid conflicts between set points in a distributed (multi-level) control system, the logic gates in each controller need to function in concert with each other. Thus, each control system functions according to its implemented “timers.”

We suggest that the transcriptional and metabolic constructs underlying goal-oriented behaviors operate as parallel sequences of a control system. We adapt the term “parallel sequences” from the field of engineering (Williams et al., 2017) to describe this multi-level regulation.

Our application of CST follows the conceptualization of Neuro-constructivism, suggesting that goal-directed behavior derives from mental representations resulting from experience-dependent development of biological constructs (Mareschal et al., 2007a,b; Sansavini et al., 2021; Farran and Scerif, 2022). Neuro-constructivism outlines how the brain subjects cognition events to aftermath processing to reach its ultimate function, goal-oriented behavior (Sirois et al., 2008).

We incorporate Neuro-constructivism concepts discriminating between regulated and non-regulated neural bases for goal-oriented behavior, founded on CST Modeling, using Boolean Algebra (Ferber, 2008). Considering that every neuronal circuit involves numerous logic gates (Ferber, 2008; von Neumann, 2017), we argue that parallel sequences, beyond simple paired-pulse facilitation, enable logic gates crosstalk at a multi-level basis reflecting a spatially encoded chrono-property of the healthy brain. This temporal consideration spans the atomic, molecular and cellular levels, covering local populations of neurons and their supporting cell types within and between the brain’s circuitry and regions. Furthermore, the conceptual parallel sequences suggest simultaneous matching of the new code with the old one, without losing previously coded information (Ferber, 2008). Based on thermodynamic, electrophysiological and neuro-molecular principles, our concept argues that within the brain, multi-level metabolic “timers” determine the time-to-action in parallel sequences and within reversible thermodynamic states.

This crosstalk between the multi-level metabolic “timers” is suggested here as parallel sequences with propagation delays of signaling at each level, in the beginning and the end of each sequence (Williams et al., 2017). The parallel sequences are apparent within each level and between levels. The efficacy of timescales is determined by the regulation of the output of these sequences, as an XOR gate. This is a function of availability of free energy at a given moment for a given internal or environmental stimulus, which in turn is limited by the given level of entropy. According to the Boltzmann Theorem, micro-states are accumulated into a macro-state of available thermodynamic free energy for a given action at a given time. Furthermore, as implied by this theorem, a macro-state exhausts itself, with a given behavioral reaction, which, however, may be reversible, when new micro-states accumulate to a new macro state, even for repeated action. In this sense, the occurrence of entropy and the exhaustion of a macro-state and a given gathered free energy for an action implies the beginning of the next macro-state. Although this claim of reversible macro-states may be understood as serial, we argue that they are computed by the different brain levels in parallel sequences, meaning numerous macro-states for emotional, cognitive and behavioral outputs at the same time.

## The model

Adding metabolic elements to CST enables viewing the brain as a thermodynamic system (Collell and Fauquet, 2015), involving a free energy-entropy matrix. Thermodynamic free energy reflects the work (useful energy) which a system can perform at constant temperature, i.e., the energy quantity available for transition or change. The corresponding temporal component is defined by the thermodynamic free energy quantities available for transition or change (Hill, 1977; Friston et al., 2006; Friston and Klaas, 2007).

According to the second law of thermodynamics, within isolated systems, entropy tends to increase over time. As the brain is not an isolated system, we suggest that the environment may alter the entropic speed in a neural network and thus change the preprogrammed availability of free energy for a behavioral action to occur (Schrodinger, 1951).

We further adopt Boltzmann’s theorem, which defines systems in thermodynamic equilibrium as capable of creating a corresponding microstate of available energy while keeping microscopic states available to the system, dependent on macroscopic limitations; meaning the system’s total level of entropy. Entropy is the probability of the tendency to proceed toward the most probable macro-state over time (Darwin, 1909; Jeffery and Rovelli, 2020). Others stated that once a system has reached its thermodynamic equilibrium, its entropy is maximized, and no further change is possible (Andelman, 1995; Villani, 2008; Brown et al., 2009; Bertolami and Gomes, 2020). In contradistinction, we posit that no true end exists for any given macro-state in a live brain throughout the life span of its neurons and supporting cell types, as each macro-state initiates the next cycle toward its following thermodynamic equilibrium. Several series of such cycles may appear in concert, such that establishing a macro-state at a certain time requires maximal total available metabolic energy for a given brain at a given moment while controlling, in parallel, the maximum number of cerebral micro-state sequences. Since there are many methods to measure entropy, we refer here to multiscale entropy (MSE), that takes times and rhythms into the account of entropy levels (Gao et al., 2015; Jaworska et al., 2018).

**FIGURE 1:**
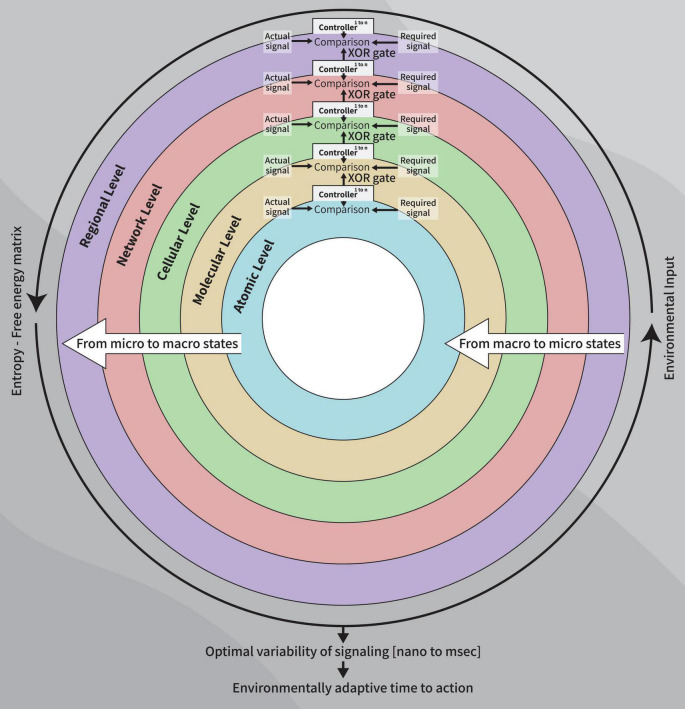
A diagram of the control systems model of the healthy clock: Parallel sequences using XOR gate as outputs between cerebral levels (controllers) resulting from micro-to macro-states dynamics, to establish the final free-energy-entropy matrix which, in turn, underlies the time-to-action to a given stimulus, at a given moment, age-appropriate.

We hypothesize that logic gates represent molecular and metabolic constructs and that XOR gates represent regulated goal-oriented behavior (Ferber, 2008). Correspondingly, XOR gates (including XNOR gates) reflect local neural networks’ excitation-inhibition balance *in vivo* (Goldental et al., 2014). Our current model (Figure 1) argues that a goal-oriented behavior depends on a temporal range of optimal signal variability based on metabolic processes, at levels spanning from atoms though molecular, cellular, network populations and inter-regional signaling, all of which operate as parallel sequences (Williams et al., 2017).

Our model involves serial parallel controllers (1 to N at each level) communicating with each other, at scales ranging from atoms through macromolecules, cells, local and large networks up to the communication between regions, all in parallel sequences (at the temporal level) from micro- to macro-thermodynamic states. To determine the final output stimulated by environmental and internal requirements (input), each controller has its own set-point for comparison processes. Set-points are intrinsic changeable capacities when the system’s environmental exposure is altered or triggered by developmental trajectories and progression in one’s age. Each unit controller consists of millions of logic gates (micro-states) whose truth tables undergo a minimization process (see Table 1, following Taub, 1982). Pruning or selection of synapses toward reaching a final XOR gate condition would hence activate a healthy and adaptive behavioral reaction (Figure 1).

**TABLE 1A:** Truth table of XOR gates output in the four parallel sequences of communication between five brain levels.

XOR output	Atom level:	Molecular level:	Cellular level:	Network level:	Regional level:
**(See reference list below)**	**A**	**B**	**C**	**D**	**E**
	0	0	0	0	0
4	0	0	0	0	1
4	0	0	0	1	0
3	0	0	0	1	1
2,3	0	0	1	0	0
2,3,4	0	0	1	0	1
2,4	0	0	1	1	0
2	0	0	1	1	1
1	0	1	0	0	0
1,4	0	1	0	0	1
1,2,3,4(*)	0	1	0	1	0
1,2,3	0	1	0	1	1
1,3	0	1	1	0	0
1,3,4	0	1	1	0	1
1,4	0	1	1	1	0
1	0	1	1	1	1
1	1	0	0	0	0
1,4	1	0	0	0	1
1,3,4	1	0	0	1	0
1,3	1	0	0	1	1
1,2,3	1	0	1	0	0
1,2,3,4(*)	1	0	1	0	1
1,2,4	1	0	1	1	0
1,2	1	0	1	1	1
2	1	1	0	0	0
2,4	1	1	0	0	1
2,3,4	1	1	0	1	0
2,3	1	1	0	1	1
3	1	1	1	0	0
3,4	1	1	1	0	1
4	1	1	1	1	0
	1	1	1	1	1

(*) represents pruned regulated functions.

**TABLE 1B:** Minimization table (*represents healthy pruning).

XOR gate output	Atom level:	Molecular level:	Cellular level:	Network level:	Regional level:
	**A**	**B**	**C**	**D**	**E**
1,2,3,4(*)	0	1	0	1	0
1,2,3,4(*)	1	0	1	0	1

Reference list for xor gate output = 1 in four parallel sequences between five levels (Tables 1A, B):

1 = sequence between A and B.

2 = sequence between B and C.

3 = sequence between C and D.

4 = sequence between D and E.

While the elements within the parallel sequences are standardly ordered when operating within the same timeframe, that timeframe depends on the range of the required signal variability (Verschure et al., 2014; Sendhilnathan et al., 2021) for a given goal-oriented behavior. To standardize the parallel sequences within a timeframe (the overall healthy range of signal variability) (Northoff, 2016), such standards may involve propagation delays at the beginning or end of the sequence, depending on its type (Williams et al., 2017). The healthy clock of each goal-oriented behavior hence comprises a fixed number of parallel sequences. Those are activated by inter-related environmental and internal stimuli (input), and processed at a given, age-appropriate moment, requiring an adaptive reaction (output).

The variables in our model determine the Truth Table for its logic gates (Table 1). Hence, pruning involves normal minimization of the original numerous logic gates (i.e., metabolic constructs) needed for the brain’s network to release a goal-directed behavior. The temporal component of these parallel sequences is determined by the ability of the macro-state to achieve a final XOR gate (regulated) condition for a healthy, goal-directed behavior. Its duration depends on the type of the environmental stimulus that requires a goal-directed action and the available inhibitory and excitatory synapses (actual value) for its adaptive activation, from all needed synapses for this action (required value).

Describing the temporal range initiated by external stimulation (input) up to the resulting behavior (output) spans the ascending (sensory) tracts, where levels ranging from atoms to regions (microstates) are triggered by environmental and internal stimuli; and the descending (motor) tracts, where goal-directed behavior reflects the entropy-free energy matrix (available macro-state at a given moment, age-appropriate).

This model implements metabolic constructs (1) at the atom level (2) ion channels at the cellular level (3) signal variability of proteins at the local network level and (4) cortical gradients for the inter-regional level.

Our model differs from models presented earlier in showing that XOR gates represent an overarching excitation-inhibition balance across multiple levels, which comprises micro- to macro-thermodynamic reversible states of the brain. Supporting this view, XOR gates have been shown to represent local neural networks’ excitation-inhibition in neural network simulations (Vogels and Abbott, 2005) and *in vivo* (Goldental et al., 2014), in accordance with our propositions.

### The clocks of the atoms

The thermodynamic perspective on neural activity and its time-dependence may involve transitions of atoms in particle diffusion models. Atomic-scale methods are defined as particle motion affected by thermal conditions, based on atomic jump frequency models. Hence, the brain complexity involved is similar to that of other diffusive phase transformations (Nasta and Soisson, 2012). The more complex picture includes self-interstitial jump mechanisms, with the same atom types occupying free locations (Martin and Soisson, 2005). Entropic (disordered) processes that affect temporal measures are detectable at the atom level (Nasta and Soisson, 2012).

### Time keepers of cell entries

Ion channels are the “time-keepers” of cell entries, recognized as pacemakers of polarization-depolarization and removal of blocking molecules (Morley et al., 1994). Ca2+ channels along dendrites recruit NMDA receptors to orchestrate the excitatory postsynaptic Ca2+ transients, amplify the spike-dependent synapse plasticity during transition and govern goal-directed behavior (Connelly et al., 2016; Tigaret et al., 2016). Ca2+ acts synchronously with recruited NMDA receptors, amplifying post-synaptic potentials linearly, independent of their specific location (Connelly et al., 2016; Tigaret et al., 2016).

Synchronous post-synaptic signaling also depends on the time delay of the depolarization for removal of NMDA Mg2+ within the small time-window of glutamate release. Therefore, the charging effects of Ca2+ signaling to nanodomain environments (Naraghi and Neher, 1997; Augustine et al., 2003; Tadross et al., 2013) can activate molecular events modulating excitation and inhibition, affecting their balanced interconnectivity (Tigaret et al., 2016). NMDA receptor channels are further characterized by slow gating, having offset decay time constant around 120 ms for the NMDA subunits NR1\NR2A, 400 ms for the NNR1\NR2B or NR1\NR2C and around 5,000 ms for the NR1\NR2D channel (Mori and Mishina, 1995). The inhibitory feedback to excitatory inputs (Vyklicky et al., 2014) is hence crucial for attaining regulated functionality toward adaptive time-to-action.

Asphyxia is caused by the collapse of the Na+K+ pump followed by Ca2+ overloading flow into the cell (Berger and Garnier, 1999). Intriguingly, this occurs at zero cellular metabolic energy (Erecińqska and Dagani, 1990; Hochachka et al., 1996; Silver et al., 1997a,b). Hence, polarization-depolarization processes reflect the thermodynamic status of the cell, based on the serial order of ions entering it. Furthermore, the Ca2+ pacemaker may protect cellular redundancy functions aimed to avoid collapse of the Na+K+ pump, cell injury and conditions of zero energy.

Given that Na+ and Ca2+ have equal molecule size, the interplay between the Na+K+ pump and Ca2+, which are all voltage-gated, forms a clock of the cell. Specifically, the complex dynamics of Ca2+ and Mg ion channels determine the propagation delay of the single neuron’s spiking and may form the granular neuronal clock and its time-to-action output.

### The clocks of neural populations

The emergence of narrowband local field potential oscillations during epileptic seizures involves completely asynchronous underlying neural activity, dysregulated in time, impairing the crosstalk between local and circuitry clocks (Gliske et al., 2017). If each neuron fires quasi-periodically, local populations of independent, completely asynchronous neurons may produce narrowband oscillations, independent of intrinsic oscillatory cells or inhibitory feedback. This unique capacity of local neurons is independent of causality pathways driven by pacemaker cells and other “time-cells” of the different brain regions. Such quasi-periodicity requires no oscillatory drive, specific network or cellular properties other than cells that are dysregulated under the fixed clock in the local neuronal sub-population that repetitively fires with continuous internal, while local, stimulation (Gliske et al., 2017). Therefore, even an uncoupled network may generate individual rhythms, implying integrative regulation between different cerebral clocks. Hence, breakdown of inhibition and high synaptic input in one location, often observed during epileptic seizures, may generate an independent narrowband oscillation and affect the behavioral output by time-dependent causality (Jaynes, 1957; Fauth et al., 2015). Signal variability at the local neural network may hence be the granular activity of a local cerebral clock, representing its time-to-action output.

### The inter-regional clocks

While past views suggested top-down and bottom-up communication (e.g., Taylor et al., 2010), recent reports showed a cortical governing role in processing internal and external stimuli. Notably, the normal clock underlying goal-oriented behavior depends on the task and environmental stimuli and is faster in sensorimotor regions and slower within associative areas (Gao et al., 2020). Correspondingly, primary sensory neurons are tightly coupled to altered environment, firing rapidly to initiate the onset and removal of the stimulus, and showing “intrinsic timescales” (Runyan et al., 2017) both at electrophysiological and molecular levels (Madrer and Soreq, 2020). In associative areas such as the prefrontal regions, neuronal activity may last many seconds when a person is engaged in cognitive processing. Quick reactivity of the sensory regions, coupled by slower reactivity in the associative regions has been recently found as resulting from “cortical gradients” which are axes connecting sensory excitation-inhibition balance and cortical regions (Huntenburg et al., 2018). These “cortical gradients” may serve as the inter-regional neural communication time-keepers. Furthermore, the time scales of cortical axes are molecular-based (Sydnor et al., 2021). Hence, our view on multi-level parallel sequences integrates the underlying time-to-action events.

### Clock genes govern the multilevel clocks

The suprachiasmatic nucleus, the central biological clock, communicates with clocks in every tissue (Astiz et al., 2019; Astiz and Oster, 2021), We argue that the central clock governs the multilevel clocks in transcriptional\translational pathways. We will show evidence for this argument by the clock genes’ (1) location\interactive function (2) molecular disorder\structural flexibility and reversibility (3) thermodynamic regulation.

### Diverse locations\interactive function

Circadian rhythms are generated, molecularly, by a feedback loop between transcriptional and translational elements. This loop includes the proteins produced by transcribed “clock genes,” and only these genes. Beyond the SCN, this feedback loop operates in many other sites within the CNS and in the periphery (Yildirim et al., 2022), including time cells in other regions (e.g., Eichenbaum, 2014; Lusk et al., 2016), suggesting that they integrate numerous timescales and level-wise clocks beyond the 24-h cycle. This has been shown across species phylogenetically, and along development starting from embryonic life ontogenetically (Ahmad et al., 2021; Poe et al., 2022). The functioning of these clocks is related to day-night cyclicity, responding to white light (Reiter et al., 2011). We suggest that this network of clocks is responsible for time-dependent (Top and Young, 2018; Patke et al., 2020) behavioral adaptation to environmental cues, beyond its photic reactivity and responsibility for the day-night entrainment.

To find out how all of these circadian clocks function together level-wise, Ahmad and colleagues have shown that the starting point at the molecular level initiates by transcriptomic processes of the clock genes, leading to the production of specific proteins affecting time scales (Ahmad et al., 2021). These proteins have been shown to affect pre- and post-synaptic plasticity in an experience-dependent manner for determining signaling within the biological clocks network (Krzeptowski et al., 2018). The interregional connectivity, in turn, is an intrinsic capacity of this clock network, ranging from the atom level, which determines the function and size of molecules, through the molecular, cellular and neural population network levels including non-coding transcripts activities.

One example is that in RNA-seq and DNA arrays to quantify the transcriptomes of 12 mouse organs over time, an organ-specific pattern was found, in which circadian rhythms of transcription were evident in 43% of functional genes in several locations in the body. The most common pattern was reflected in early morning and evening transcriptional peaks. Compared to non-rhythmic genes, oscillating genes were longer, closer to each other and revealed more alternatively spliced forms. There was considerable coactivation of genes all over the body. Over 1,000 noncoding RNAs (ncRNAs) also showed rhythmic expression, in similar percentages as genes coding for proteins.

A second example is that clock genes (Okamura, 2004) engage in polymorphic interactions with multiple non-coding RNA molecules, including but not limited to the recently re-discovered small transfer RNA fragments (tRFs), e.g., (Winek et al., 2020), or long-non-coding RNAs (Mosig and Kojima, 2022). These and other regulatory RNAs regulate the molecular clocks in both the SCN and the periphery (Mendoza-Viveros et al., 2017; Zhu and Belden, 2020) and in every tissue, which in turn affects signal variability of the single cell, within neural networks and inter-regionally.

A third example is that the product of the LUX clock gene, beyond its negative autoregulatory feedback loop, regulates gene expression of another element in the transcriptional loop, PSEUDO RESPONSE REGULATOR9 (PRR9).

A fourth example is that an interaction has been described between Per1 interacting protein of the suprachiasmatic nucleus (PIPS), a ∼180-kDa protein, and the clock gene mouse Per1 (mPer1). The SCN is one of the main sites of PIPS expression (Matsuki et al., 2001).

We further adopt the previously reported concept of a feedback loop involving transcriptional and translational mechanisms, level-wise, which underlies this complexity of time regulation by clock genes (Ahmad et al., 2021). In this sense, we describe the larger reactivity of the known central and peripheral biological clocks and show their signaling complexity as a “cross-talk” of time scales regulating each other for a healthy goal-oriented behavior.

### Molecular disorder\structural flexibility and reversibility

Intrinsically Disordered Proteins (IDPs) and Intrinsically Disordered Protein Regions (IDPRs) are proteins with a variety of steric conformations, found in macromolecular organization. Protein disorder and conformational plasticity are important for providing tuneability and stochasticity in many biological systems (Oldfield and Dunker, 2014). Others showed that disordered proteins are essential for the clocks’ adaptive alterations to environmental cues (Pelham et al., 2020). We thus emphasize the role of this previously shown intrinsic flexibility of the central and peripheral clocks’ functioning for a given action, at a given time. Specifically, we argue that reacting to given stimuli with available macro-states may achieve this action using feedback loop mechanisms level-wise (Pelham et al., 2020). In this sense, the molecular disorders observed in clock genes, and the aligned flexibility of their structure and function, suggest their role in shorter and longer time scales than the 24-h cycle.

### Thermodynamic regulation

From a thermodynamic perspective, clock genes are involved in protein production and their related release of free energy. The interaction between clock genes is also a source of energy release. Clock genes are able to quantify irreversible entropic processes and pathways (Vallée et al., 2018).

Free energy sources originate from glucose and its synthesis (Deitmer et al., 2019). One major derivate is lactate. It is transferred between glycolytic and oxidative cells, both in normal and hypoxic conditions (Deitmer et al., 2019).

Contrary to cellular energy suppliers, interactions between circadian rhythms, WNT/beta-catenin pathway and PPAR gamma interfere significantly with thermodynamic equilibrium (Vallée et al., 2017).

Different protein isoforms interact with each other. This releases energy, useful for metabolism, and consumes free energy (Vallée et al., 2017; Deitmer et al., 2019). Additionally, lactate conversion is reversible, allowing cells to either produce or consume lactate, depending on their metabolic profile (Deitmer et al., 2019). From a thermodynamic perspective, the final available quantity of free energy for a given behavior at a given moment in a given environmental condition, also depends on the levels of entropy.

The entropy production rate determines irreversible processes. Metabolic enzymes show changes in thermodynamic properties, following dysregulation of the canonical WNT/beta-catenin pathway. Upregulation of this pathway results in aerobic glycolysis and alters the thermodynamics of important enzymes. Downregulation of this pathway produces oxidative stress and apoptosis (Vallée et al., 2018). These conditions show the crucial role of clock genes for free energy by their control of the Redox systems and oxygen scavengers, e.g., melatonin, reducing entropy (Marseglia et al., 2015; Vallée et al., 2017).

Thus, we suggest that to allow transport of proteins to carry information to and from clock genes effectively, through post-synaptic activation, the body’s circadian clock and its oscillations organizes the timing of gene-expression to initiate and control underlying multi-level parallel processes for goal directed behavior.

### Model implications: clock genes as master controllers of time and free energy

A Master controller, in control systems, is defined as a receiver of the all the dependent multi-level controllers’ data, and a transmitter of desired values (set points) to all the multi-level controllers, for reprocessing.

According to our model, initiation of time-to-action is apparent in the level of protein isoforms of the clock genes for ion entries into the cell, cellular activation and cell protection. This initiation, in turn depends on the smoothness of progression through XOR gates from gene expression to the molecular clocks and then toward a final differential cell firing rate which is consistently subjected to multiple regulators. This reflects, in our view, optimal ranges of signal variability in healthy adults. Specifically, the parallel processes, level-wise, continue from this initiation to neural population networks and interregional pathways with feedback loops back to clock genes. We suggest that as clock genes exhibit flexible structure and function, they are enabled to work as receivers (input), not only as initiators and transmitters (output). For example, the input: NMDA receptor regulates the circadian clock (Hearn et al., 2023) whereas output: clock genes are involved in the pathogenesis of epilepsy (Wu et al., 2021; Bazhanova, 2022). We argue that these clock genes’ capacity stays beyond their photic reactivity and for shorter and longer timescales than 24 h cyclicity. This presents the clock genes as master controllers, calculating free-energy and time-to-action of behavioral manifestations.

We suggest that the clock genes use available signaling of excitation-inhibition balance. We argue that inhibitory signals and their metabolic gradients also accumulate in the controlling calculator at the same extent as the excitatory ones. Thus, the healthy regulated time-to-action is determined by the resultant calculated excitation-inhibition balance and its available free energy vs. entropic conditions. This balance, in turn, determines the “open” neural pathways for a healthy behavioral action to be actualized.

We also argue that these excitatory and inhibitory signaling feedback loops enable the clock genes to calculate free energy with a given status of entropy which in turn results in time-to-action estimation on the transcriptional level. The XOR gates regulating behavioral action are also calculated in the SCN and in the periphery by the clock genes’ interactions. The calculation of time and free energy for a required behavioral action further depends on protein transporters. This is also due to the diverse locations, functions and structures of the clock genes. The reversibility of macro-states implied in the Boltzmann theorem is seen in the reversibility of lactate production and in the Redox and oxygen scavengers substrate, determining the free energy spectra for a required action. From a thermodynamic perspective, the quantity of available free energy determines the time required for a behavioral action. The calculated time-to-action for an outcome of optimal signal variability is derived from the calculation of free energy. Clock genes, as master controllers, receive the input of the inhibitory-excitatory balance status of the multilevel clocks and calculate the free energy for an action accordingly. A regulated environmentally adaptive action may be actualized if the clock gene calculations are based on excitation-inhibition balance dictating feedback loops of parallel processes level-wise signaling.

## Derived hypotheses and molecular predictions

Our model may be adapted to either narrower or broader prisms\spectra of neural and brain functioning by defining distinct numbers of levels. This may involve aligned Truth Tables and their minimization for XOR gate output and may generate further hypotheses. Our overarching hypothesis relates to regulating the excitation-inhibition balance from atoms through molecular, cellular network and inter-regional levels. We investigate the interim pathways from the genetic quasi-deterministic levels of time-to-action to experienced-modified signal variability and its optimal range (Goldstein Ferber et al., 2022). This could be examined by in-vivo tests of the healthy signal variability range and its XOR gate functionality. Such studies may open novel opportunities for more accurate and reliable diagnoses and improved prevention and treatment of psychopathologies.

The abundant mental disorders in humans are presumably rooted in an evolutionary environmental-biology mismatch, and may predictably interrupt XOR processes. Nevertheless, pinpointing the biological mechanisms that regulate the corresponding behaviors and their mental consequences is challenging. Recently, a novel class of non-coding RNA molecules, called transfer RNA fragments (tRFs) was identified as playing critical roles in timing brain activities. However, how are these factors dysregulated in disease, what are the molecular mechanisms through which tRFs act in response to stress and to what extent are these mechanisms evolutionarily conserved remains unknown.

Gene regulation is a fundamental process that dictates which genes of a cell are actively expressing functional gene products, including RNA and proteins, at any given time (Soreq, 2015). Even though each brain cell contains the same genetic code, different genes are expressed in each cell context-dependently, through multi-step regulatory processes. These events define the identity and function of a cell and can be subject to change, depending on the internal and the external environments experienced during the life course and mental state of an individual.

Non-coding RNAs (ncRNAs) are biological molecules regulating gene expression and cellular function. Historically, they have been thought to be ‘junk’ RNA, originating from errant transcription, or breaking down of no longer needed RNA molecules. However, some ncRNA classes were shown to regulate the expression of genes in a human disease-dependent manner. Recently re-discovered among ncRNAs are transfer rNA fragments (tRFs), which originate from the targeted fragmentation of tRNAs that carry amino acids to the ribosome to enable protein synthesis. Recent findings showed that tRFs are not random fragmentation products of cellular metabolism, but rather a novel, understudied class gene expression regulators, whose biogenesis is controlled by precise and conserved site-specific cutting mechanisms (e.g., Winek et al., 2020, 2021) in human health and disease.

While tRFs have recently been shown to have functional features similar to those of microRNAs (miRs), it is still unknown if they regulate behavior. Several tRFs contribute to embryonic development and neuronal function, with their abundance changing with age (Karaiskos et al., 2015; Cao et al., 2021; Paldor et al., 2022), including tRFs originating from the tRNA LysTTT. While their complex impact on brain-to-body functioning is largely unresolved, small regulatory RNAs are rapidly acquiring wide recognition as global controllers of regulatory processes at large and may emerge as controllers of the above-described brain clocks. Thus, tRFs may emerge as the initiators (the “onset”) of time-dependent parallel sequences ranging from atom levels through molecular, cellular, local network and interregional signaling. Supporting this notion, tRFs are sensitive to environmental stimuli and able to trigger the available metabolic processes (e.g., Winek et al., 2020) termed here as thermodynamic macro-states for an action to be performed. A further prediction, based on the findings on the regulatory role of tRFs (e.g., Dubnov and Soreq, 2023), is that tRFs will facilitate the regulatory output of *in vivo* XOR gates within the parallel sequences at the different levels determining time-to-action functionality.

One possible design to test our model could be to concurrently sample event-related H1-magnetic resonance spectroscopy (MRS) and electroencephalography (EEG) as in Lally et al. (2014), across different environmental stimuli and at different ages. The MRS can provide measures on the levels from atoms through molecules to cells and the EEG can detect local neural networks and inter-regional connectivity.

## Discussion

We view the brain clocks of time-to-action as a control system dependent on the rhythms of regulated metabolic constructs for its healthy and timely signaling. The entire range from atomic, molecular, cellular, local circuitry and inter-regional network levels operates in parallel, computing concomitantly sequential signals from the lowest atomic level up to the highest level of inter-regional neurite functionality. This sequential simultaneous signaling represents combined parallel processing for the final measure of time-to-action. We characterize healthy time-to-action by its excitation-inhibition balance at each level being within an optimal range of the individual’s chrono-properties. Clock genes are designed for enabling initiation of these parallel processes, calculations of free energy and derived optimal time-to-action.

The brain is an open thermodynamic system and its levels of entropy increase or decrease by interacting with the environment, which may be under-stimulating, stressful or regulatory, thus determining the sum of available synapses. Additional factors including age, preprogrammed developmentally appropriate steps (Ferber et al., 2022) and previous encounters affect pruning and experienced-shaped behaviors (Sirois et al., 2008), and determine available synapse numbers compared to the required number for a regulated, age-appropriate time-to-action at a given moment.

In sum, the clocks operate at atoms, cells, local neural populations and inter-regional levels. The healthy clocks of time-to-action regulate XOR logic gates communicating between micro- and macro-states. Imbalanced excitation-inhibition, represented by deviations from XOR gates to other types of logic gates, shows suppressed healthy pruning. Finally, experience-shaped neural structures and their macro-state in a given environmental exposure determine the underlying parallel sequences of clocks required for an action.

## Data availability statement

The original contributions presented in this study are included in the article/supplementary material, further inquiries can be directed to the corresponding author.

## Author contributions

SGF was the leading author. All authors contributed substantially to the different versions of the manuscript and to its final version.
